# Influence of the Synthetic Cannabinoid Agonist on Normal and Inflamed Cartilage: An In Vitro Study

**DOI:** 10.3390/biom13101502

**Published:** 2023-10-10

**Authors:** Jiangyinzi Shang, Sophie Hines, Meagan J. Makarczyk, Hang Lin, MaCalus V. Hogan, Alan Yan

**Affiliations:** 1 Department of Orthopaedic Surgery, University of Pittsburgh School of Medicine, Pittsburgh, PA 15213, USA; jiangyin@pitt.edu (J.S.);; 2 Department of Bioengineering, University of Pittsburgh Swanson School of Engineering, Pittsburgh, PA 15213, USA; 3 McGowan Institute for Regenerative Medicine, University of Pittsburgh School of Medicine, Pittsburgh, PA 15213, USA; 4 MBA Kaufmann Medical Building, 3471 Fifth Avenue, Suite 1011, Pittsburgh, PA 15213, USA

**Keywords:** cannabis, marijuana, endocannabinoid, cannabinoid receptor, osteoarthritis, cartilage

## Abstract

Medical marijuana (versus Marijuana derivatives) has been reported to possess analgesic, immunomodulatory, and anti-inflammatory properties. Recent studies in animal models of arthritis showed that cannabinoids, a group of compounds produced from marijuana, may attenuate joint damage. However, whether marijuana byproducts can suppress osteoarthritis (OA)-associated cartilage degradation has not been previously reported. In this study, human chondrocytes were isolated from healthy articular cartilage, expanded in vitro, and subjected to pellet culture in a chondrogenic medium to form cartilage tissues. We first examined the influence of marijuana byproducts on normal cartilage by treating chondrocyte-derived tissues with a synthetic cannabinoid agonist, Win-55,212-2 (Win), at different concentrations ranging from 0.01 to 10 µM. After treatment, the tissue phenotype was assessed using glycosaminoglycan (GAG) assay and real-time PCR. Next, cartilage tissues were pre-treated with interleukin-1β (IL-1β) to generate an inflamed phenotype and then cultured with Win to assess its therapeutic potential. The results showed that at concentrations lower than 1 µM, Win treatment did not significantly impair chondrocyte growth or cartilage formation capacity, but at a high level (>10 µM), it remarkably suppressed cell proliferation. Interestingly, under the condition of IL-1β pre-treatment, Win was able to partially preserve the cartilage matrix and decrease the production of interleukin-6, although the protective effect was mild. Taken together, our results indicated that the variable effects of Win on chondrocytes occur in a concentration-dependent manner. Whether cannabinoid derivatives can be used to treat cartilage degradation or can alter other structural changes in OA deserve further investigation.

## 1. Introduction

Cannabis (marijuana) was first described by the legendary Chinese emperor Shen Nong, dating back to the 18th century BCE [1], and has been used throughout history for medicinal use as well as recreational purposes [2]. In the United States, cannabis was first used to relieve pain and treat spasticity. Currently, marijuana has been legalized in 37 states and the District of Columbia for medical use. Since the implementation of medical cannabis laws, 18 states have legalized the use of marijuana by adults for recreational and medical purposes. Medical marijuana is a term for derivatives of the Cannabis sativa plant that are used to ease symptoms caused by certain medical conditions. Medical marijuana is also known as medical cannabis [2]. There is a growing body of scientific interest and evidence regarding the use of marijuana to treat various medical conditions. Among them, research interests are focused on chronic pain control and the potential use of marijuana derivatives as alternatives to reduce opioid usage, which are the most commonly cited methods for pain management [3]. There are many ongoing trials for which the medical effects of cannabinoids are being explored, including orthopedic research [4]. For example, the Cannabinoid Profile Investigation of Vaporized Cannabis in Patients with Osteoarthritis of the Knee study was initiated in 2014 (NCT02324777), and another clinical trial is evaluating combinations of cannabinoids, opioids, and benzodiazepines for their pain-relieving effects in a small number of patients with osteoarthritis (NCT03098563).

Cannabinoids function through the endocannabinoid system in our body, which is composed of endocannabinoids, receptors, and other associated endogenous ligands. The endocannabinoid system is involved in many homeostatic processes, including motility of the gastrointestinal tract, feelings of hunger, perception of pain, and immunity [5,6,7,8]. The two main targets of the endocannabinoid system are classical cannabinoid receptor 1 (CB1) and cannabinoid receptor 2 (CB2). CB1 is defined as the neuronal cannabinoid receptor that regulates neurotransmission and multiple peripheral functions [9,10]. CB2 is considered non-neuronal, regulates immune and inflammatory pathways, and mainly affects the immune system around B cells, natural killer cells (NK cells), and T cells [11,12]. Physiologically, both CB1 and CB2 are activated by endogenous fatty-acid-derived ligands or specific peptides [13], but they can also be activated by exogenous cannabinoids, mediating the impact of cannabis on humans. 

Currently, the direct influence of cannabinoids on cartilage or chondrocytes is not clear. Estery et al. reported that in bovine articular chondrocytes, the non-selective synthetic cannabinoid agonists HU-210 and Win 55,212-2 (Win) substantially inhibited interleukin (IL)-1α-stimulated proteoglycan and collagen degradation. Win also inhibited the expression of inducible nitric oxide synthase (iNOS) and cyclooxygenase-2(COX-2) and caused the activation of the nuclear factor kappa-light-chain-enhancer of the activated B cell (NF-κB) pathway, which is a vital pathway for osteoclasts [14]. In another relevant study, Win mesylate was shown to suppress interleukin-1β-induced expression of matrix metallopeptidase (MMP)-3 and -13 in human chondrocytes in 3D alginate bead cultures [15]. However, this study was performed using a monolayer culture condition, which does not represent the three-dimensional environment in which chondrocytes reside within cartilage. In addition, the effects of Win on healthy cartilage tissues were not examined.

In this study, we aimed to examine the influence of Win on both healthy and inflamed chondrocytes in the context of three-dimensional culture, a more physiologically relevant culture system than previous models. Specifically, human chondrocytes were isolated from healthy articular cartilage, expanded in vitro, and subjected to pellet culture in a chondrogenic medium to form cartilage tissues. We first examined the influence of marijuana byproducts on normal cartilage by treating chondrocyte-derived tissues with a synthetic cannabinoid agonist. After treatment, the tissue phenotype was assessed using glycosaminoglycan (GAG) assay and real-time PCR. Next, cartilage tissues were pre-treated with interleukin-1β (IL-1β) to generate an inflamed phenotype and then cultured with Win to assess its therapeutic potential.

## 2. Materials and Methods

### 2.1. Cell Isolation and Expansion

With the approval from the University of Pittsburgh Committee for Oversight of Research and Clinical Training Involving Decedents (#878) on 11 July 2019, cartilage tissues from the knee joints were harvested from deidentified donors without joint diseases. To isolate chondrocytes, fresh articular cartilage tissues were first rinsed with high-glucose Dulbecco’s Modified Eagle Medium (DMEM, Gibco/Thermo Fisher Scientific, Waltham, MA, USA) containing 2 × antibiotics-antimycotics (Life Technologies, Carlsbad, CA, USA), cut into ~1-mm^3^ pieces, and then digested in 10 mL/g wet weight cartilage dissection medium with collagenase type II (Worthington Biochemical Corporation, Lakewood, NJ, USA) at 1 mg/mL (*w/v*) for 16 h on a shaker at 37 °C.

The mixture was then passed through a 70 μm cell strainer to collect chondrocytes. The isolated cells were seeded in tissue culture flasks at a density of 1 × 10^4^ cells/cm^2^ and maintained in a growth medium (DMEM containing 10% fetal bovine serum (Invitrogen, Carlsbad, CA, USA) and 1% antibiotic-antimycotic). After the cells were fully attached to the culture substrate (usually after 7 days), the medium was changed every 3 days until the cells reached 70–80% confluency. The cells were detached using trypsin/EDTA (Gibco, Thermo Fisher Scientific) and passaged. Since this study needed to use a large number of cells, Passage 1 (P1) chondrocytes from eight donors were pooled (Supplementary Table S1). Passage 2 chondrocytes were used in all the experiments below.

### 2.2. Materials 

Win 55,212-2 mesylate salt ≥98% (Sigma-Aldrich, St. Louis, MO, USA), dimethyl sulfoxide (DMSO, Life Technologies, Carlsbad, CA, USA), transforming growth factor-β3 (Peprotech, Rocky Hill, NJ, USA), insulin-transferrin-selenium supplement (Invitrogen), antibiotic-antimycotic (Invitrogen), Quant-iT PicoGreen dsDNA reagent and kits (Invitrogen), TRIZOL reagent (Invitrogen), SuperScript VILO cDNA synthesis kit (Invitrogen), Applied Biosystems Power SYBR Green PCR Master Mix (Invitrogen), and 10% buffered formalin phosphate solution (Fisher Chemical, Fair Lawn, NJ, USA) were used. CellTiter 96 AQueous One Solution cell proliferation assay (MTS) and Accumax cell dissociation solution were purchased from Promega (Madison, WI, USA) and Innovative Cell Technologies (San Diego, CA, USA), respectively. 

### 2.3. Proliferation Assay

Pooled chondrocytes at passage 2 were seeded in a 24-well culture plate at 5000 cells/well and treated with different concentrations of Win (0.01, 0.1, 1, 5, 25, and 50 µM) in the growth medium. A stock solution of Win in DMSO with a concentration of 50 mM was prepared. Subsequently, the Win stock was diluted into different concentrations, with the final DMSO concentration below 0.1%. On days 0, 1, 3, and 7, the metabolic activity of the cell culture was measured using MTS. Briefly, 300 µL of the chondrogenic medium with 60 µL of the MTS solution were added into each well and co-cultured with chondrocyte-laden hydrogels for 2 h. The formazan dye produced by viable chondrocytes was quantified via colorimetric measurements at 490 nm using an ELISA microplate reader (BioTek Synergy H1, Winooski, VT, USA). Based on the data from the MTS assay, the half-maximal inhibitory concentration (IC50) was calculated. 

### 2.4. Pellet Culture

Conventional chondrocyte pellet cultures were used to create cartilage tissue in vitro. Briefly, 250,000 chondrocytes were resuspended in the chondrogenic medium (high-glucose DMEM, 1% antibiotic-antimycotic, 1% insulin-transferrin-selenium supplement, 100 nM dexamethasone, 50 μM ascorbic acid, 23 μM L-proline, and 10 ng/mL transforming growth factor β3), and pellets were formed on a 96-well conical-bottomed plate via centrifugation for 10 min at 300× *g*. The medium was changed every 2 to 3 d. After differentiation for 14 d, pellets were treated with different concentrations of Win (0.01, 0.1, or 1 µM) for another 48 h and then harvested on day 16.

Next, to generate an inflamed phenotype, the proinflammatory cytokine IL-1β (10 ng mL^−1^; PeproTech, Rocky Hill, NJ, USA) was introduced into the medium for 2 days (day 14–day 16), during which transforming growth factor β3 and dexamethasone were removed from the medium. Subsequently, pellets were treated with different concentrations of Win (0.01, 0.1, or 1 µM) for another 48 h and then harvested on day 18.

### 2.5. RNA Isolation and qRT-PCR

Total RNA was obtained by homogenizing the samples in Qiazol Lysis Reagent, purified using an RNeasy Plus Mini Kit (Qiagen, Hilden, Germany), and quantified spectroscopically using a Nanodrop 2000c Spectrophotometer (Thermo Fisher). Reverse transcription was performed utilizing SuperScript™ IV VILO™ Master Mix, and qRT-PCR was performed on the Applied Biosystems real-time PCR system using SYBR Green Reaction Mix. Relative expressions of SRY-box transcription factor 9 (*SOX9*), collagen type II alpha 1 chain (*COL2*), aggrecan (*AGG*), alkaline phosphatase, MMP-13, collagen type X (*COL10*), ADAMTS4 (*ATS4*), cannabinoid receptor 1, and cannabinoid receptor 2 (Table S2) were calculated and analyzed using the ΔΔCT method. Human ribosomal protein L13a (*RPL13a*) was used as housekeeping gene control.

### 2.6. Safranin-O/Fast Green Staining

The pellets were fixed in a 10% buffered formalin phosphate solution overnight at 4 °C and dehydrated using an increasing ethanol gradient. After soaking in xylene for 2 h and then in liquid paraffin overnight, the samples were embedded in paraffin. The blocks were sectioned at a 6 μm thickness using the RM 2255 Fully Automated Rotary Microtome (Leica Biosystems, Buffalo Grove, IL, USA). The slides were then dried and subjected to Safranin-O/Fast green counterstaining (Sigma-Aldrich). Nuclei were stained with hematoxylin (Sigma-Aldrich). After staining, slides were mounted with a Limonene-Mount mounting medium (Electron Microscopy Sciences, Hatfield, PA, USA) and then covered, and histological staining was performed using a microscope equipped with a color digital camera (Nikon Eclipse E800).

### 2.7. Sulfated Glycosaminoglycan (sGAG) Assay

Samples were enzymatically digested at 60 °C overnight in a papain solution (125 μg/mL papain, 50 mM sodium phosphate buffer, 2 mM N-acetyl cysteine, pH 6.5). The GAG content was quantified using a dimethyl methylene blue dye-binding assay (Blyscan, Biocolor, Antrim, UK) according to the manufacturer’s instructions. Total DNA content was measured using the Picogreen dsDNA assay.

### 2.8. Quantification of the Enzyme-Linked Immunosorbent Assay (ELISA)

The medium from each well was sampled after treatment with Win for 48 h. Samples were centrifuged at 2100 rpm for 10 min, and the cell-free culture media supernatants were stored at −80 °C until analysis was conducted using a Quantikine human interleukin-6 (IL-6) ELISA kit (R&D Systems, Minneapolis, MN, USA) according to the manufacturer’s instructions.

### 2.9. Statistical Analysis

Statistical analysis was carried out using GraphPad Prism 9 (GraphPad, San Diego, CA, USA). All data are presented as mean ± standard deviation for analysis. One-way or two-way analysis of variance (ANOVA) was conducted for multi-comparison between groups. Tukey’s post hoc multiple comparisons test was selected as the posttest method for ANOVA. *p* values less than 0.05 were considered statistically significant.

## 3. Results

### 3.1. Win (<1 µM) Does Not Adversely Affect Human Chondrocyte Proliferation

**Figure 1:**
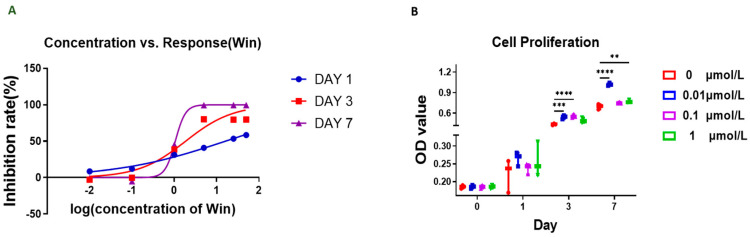
MTS assay was used to assess the influence of Win on chondrocyte proliferation. (**A**) The inhibition rate of Win at different concentrations was calculated based on the absorbance in the MTS assay. (**B**) MTS assay performed on days 0, 1, 3, and 7 of the chondrocyte culture. ** *p* < 0.01, *** *p* < 0.001, **** *p* < 0.0001. N = 3.

We first tested the effect of Win treatment on the proliferative capacity of chondrocytes at a final concentration of 0.01–50 µM. Cell numbers on days 1, 3, and 7 were measured using MTS. As shown in Figure 1A, the half-maximal inhibitory concentration of Win on chondrocyte proliferation was between 1 and 16 µM (16.1, 1.85, and 1.06 µM for days 1, 3, and 7, respectively). We further examined the response of the chondrocytes to Win by including more replicates (Figure 1B). In all tested concentrations (0.01, 0.1, and 1 µM), Win did not impair the proliferation of chondrocytes in the two-dimensional culture. In fact, at 0.01 µM, Win displayed a mild beneficial effect on chondrocyte growth. 

### 3.2. Win (<1 µM) Does Not Impair In Vitro Cartilage Formed by Human Chondrocytes

**Figure 2:**
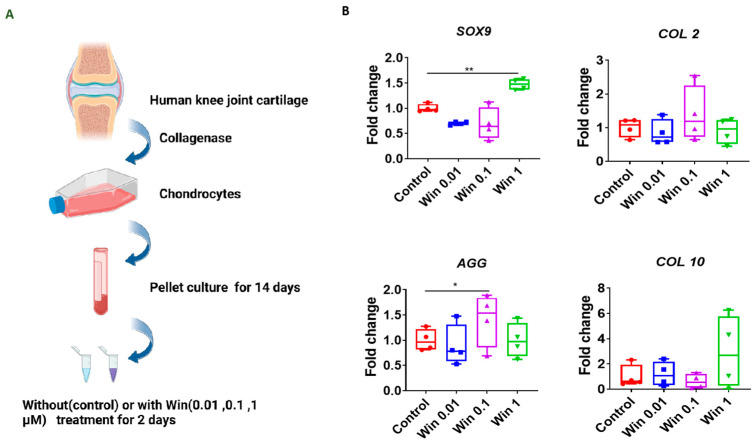
Assessment of the influence of Win on normal cartilage pellets. (**A**) Schematic to show the experimental process. The figure was created with BioRender.com. (**B**) Relative gene expression levels in cartilage pellets treated with different concentrations of Win (0.01, 0.1, and 1 µM). Data in the Control group, which was not treated with Win, were set as 1. * *p* < 0.05; ** *p* < 0.01. N = 4.

Next, we examined the impact of Win on cartilage created by human chondrocytes. After being maintained in a chondrogenic medium for 14 days, newly formed cartilage pellets were cultured with Win for 2 days (Figure 2A). Win treatment significantly increased the expression of *Sox9* or *AGG* at 1 or 0.1 µM, respectively (Figure 2B). In all other tested chondrogenic and proinflammatory cytokine genes, including *COL2* and *COL10,* some differences were noted when chondrocyte pellets were treated with Win. However, no statistically significant differences were observed. 

**Figure 3:**
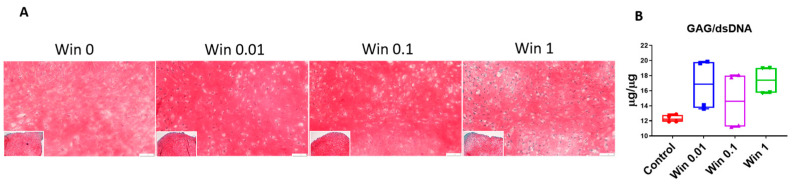
Safranin-O staining (**A**) and GAG assay (**B**) to examine cartilage pellets treated with Win at different concentrations (0–1 µM). Bar = 50 µm. N = 4 biological replicates.

We used safranin-O staining and a GAG assay to assess cartilage matrix deposition. As shown in Figure 3, at all tested concentrations, Win displayed a capacity to promote GAG generation, although no statistical difference was found. In summary, Win, at concentrations lower than 1 µM, did not adversely impact the normal cartilage tissues in vitro created by human chondrocytes. 

### 3.3. Win (<1 µM) Cannot Fully Reverse the Changes of Gene Expression Induced by IL-1β in Cartilage Tissues

**Figure 4:**
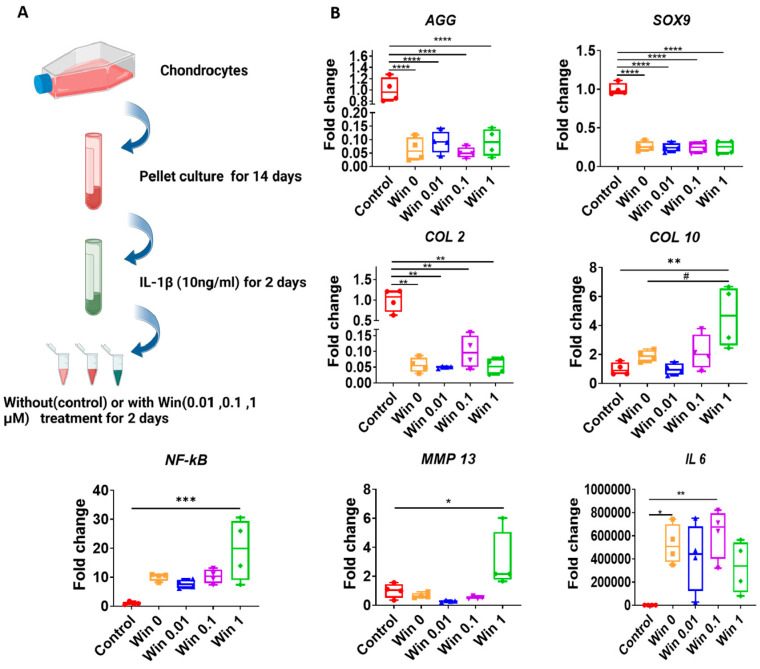
Assessment of the influence of Win on inflamed cartilage pellets. (**A**) Schematic to show the experimental process. The figure was created with BioRender.com. (**B**) Relative gene expression levels in IL-1β-pre-treated cartilage pellets that were co-cultured with different concentrations of Win (0, 0.01, 0.1, and 1 µM). The group without treatment of Win or IL-1β served as the Control (set as 1). Groups treated with Win at concentrations of 0, 0.01, 0.1, and 1 µM were all pre-treated with IL-1β. * *p* < 0.05; ** *p* < 0.01; *** *p* < 0.001; **** *p* < 0.0001; # *p* < 0.05. N = 4.

Next, we explored the therapeutic potential of Win when the cartilage was in an inflammatory environment, a condition that is often observed in joint injury and arthritis. When comparing the control and Win 0 groups (Figure 4A), IL-1β treatment significantly suppressed the expression of anabolic genes, such as *SOX9, COL2*, and *AGG*, and conversely increased the expression of proinflammatory cytokines, such as *NF-kB*, *IL-6*, and *MMP-13* (Figure 4B). These results indicated the successful generation of inflammation in the cartilage tissues. 

In all groups that were co-treated with Win, the IL-1β-induced gene expression changes in cartilage tissues were not reversed. Moreover, at the concentration of 1 µM, Win induced a higher expression of catabolic genes, including *COL10*, *NF-kB*, and *MMP-13*.

### 3.4. Win (0.01 µM) Preserves GAGs in IL-1β-Pre-Treated Cartilage Tissues 

**Figure 5:**
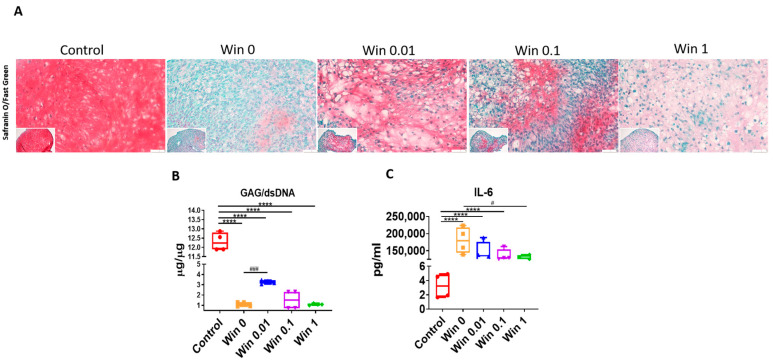
Safranin-O staining (**A**) and GAG assay (**B**) to examine cartilage pellets treated with Win at different concentrations (0–1 µM) under an inflamed condition. Bar = 50 µm. (**C**) ELISA to examine IL-6 levels in the conditioned medium from different groups. The group that did not undergo Win or IL-1β treatment served as the Control (set as 1). Win 0, 0.01, 0.1, and 1 groups were all pre-treated with IL-1β.; **** *p* < 0.0001; # *p* < 0.05; ### *p* < 0.001. N = 4.

We next assessed the GAG deposition using safranin-O (Figure 5A). Surprisingly, at a low concentration of 0.01 µM, Win treatment was able to preserve more GAGs than in the untreated group. This result was further confirmed using the GAG assay (Figure 5B). Finally, we measured IL-6 levels in the condition media from different groups (Figure 5C). Interestingly, at a high concentration of 1 µM, Win slightly reduced the IL-6 concentration from approximately 223.82 ng/mL (untreated) to 112.67 ng/mL. However, its potential therapeutic value is limited since the IL-6 level in normal cartilage is approximately 3 pg/mL. 

## 4. Discussion

In this study, we found that Win, at low concentrations, did not have a negative influence on de novo cartilage from in vitro cultured chondrocytes. However, when chondrocyte-derived cartilage was in an inflamed state induced by IL-1β, Win displayed a protective effect, but that was not able to fully reverse the damage caused by IL-1β. 

Given the fact that marijuana is being consumed by an increasing number of people, it is imperative to examine the potential health impact of such a trend, especially when used for medical purposes. The first aim of this study was to examine how marijuana-derived products affect cartilage health. It should be noted that many different types of byproducts are deposited in the body after marijuana use. In a review article, cannabinoids were found to be one of the main modulators of an immune response [16]. Therefore, we selected cannabinoids as the primary research target. Win has been previously shown to function through binding cannabinoid receptor 1 and cannabinoid receptor 2 [17]. Both receptors could also be activated in painful diseases, such as arthritis, gout, and musculoskeletal trauma [18]. Pre-clinical research on the endocannabinoid system revealed that mRNA and proteins of CB1 and CB2 were identified in the synovial tissues of osteoarthritis and rheumatic arthritis patients [19]. Moreover, Win reduced the levels of IL-6, -8, -18, and TNFα released during SARS-CoV-2 infection in heart cells and attenuated cytotoxic damage measured via the release of lactate dehydrogenase [20]. A previous study demonstrated that intrathecally co-administered Win and bupivacaine produced synergistic antinociceptive interactions in a rat formalin test [21]. Another in vivo study reported that Win alleviated acute lung injury in mice with sepsis via the suppression of glycolysis and differentiation of M1 alveolar macrophages stimulated by lipopolysaccharide (LPS) [22].

One previous study confirmed the expression of both CB1 and CB2 receptors in human chondrocytes [9]. However, Win can also function as an independent receptor. For example, at a concentration lower than 2 μM, Win decreased the production of IL-6, -8, and MMP-3 via a Transient receptor potential vanilloid 1- and Transient receptor potential ankyrin 1-dependent pathway without the need for cannabinoid receptors type 1 and 2 [23]. 

In an early study, Winklmayr et al. [24] showed that cannabidiol was toxic to cultured human chondrocytes when the concentration was higher than 5 µM, which was similar to our findings of the half-maximal inhibitory concentrations of Win from day 1 to day 7, being between 1 and 15 µM. In fact, in the studies testing mantle cell lymphoma or human cholangiocellular carcinoma cell line HuCC-T1 [25], it was reported that cannabinoids did not express noticeable cytotoxicity under the dosage of 5 µM. Human synovial fibroblasts also survived for 48 h after the Win treatment at the dosage of 10 µM [23]. In the same study by Winklmayr et al., higher concentrations of cannabinoid receptor type 1 (>10 µM) were tested. The physiological relevance remained unclear. Currently, there are no data demonstrating the cannabinoid concentration in the serum or synovial fluid after marijuana use. 

An attractive research topic related to cannabinoids is their anti-inflammatory effects. In an in vitro study that tested fibroblasts isolated from patients with osteoarthritis or rheumatoid arthritis, Win, in low concentrations, exhibited anti-inflammation effects [23]. In addition, a recent study by Karuppagounder et al. reported that cannabinoid oil and cannabigerol oil ameliorated pain and inflammation [26]. Reports also showed improved gait and locomotive activity in osteoarthritic mice that were induced by surgical destabilization of the medial meniscus [27]. This study indicated the inflammation-modulating function of cannabinoids; however, it was reported that cannabinoid oil alone did not significantly suppress cartilage loss. In a previous study, Jin et al. fabricated cannabidiol-loaded poly(lactic-co-glycolic acid) copolymer nanoparticles and tested their potential in LPS-treated chondrocytes. The results demonstrated the capacity of cannabinoids to suppress the expression of IL-1β, IL-6, TNFα, and MMP-13. However, pathological changes due to LPS treatment showed no proof of full recovery in chondrocytes [28]. These findings were consistent with our in vitro work, which showed the protective influences of cannabinoids but that they could not completely reverse cartilage degradation induced by IL-1β. In our recent study using a microphysiological system, we reported a new strategy that combines anti-inflammation and chondroinduction, which considerably preserves the cartilage matrix in an osteoarthritic environment [29]. Therefore, to further enhance the therapeutic efficacy during osteoarthritis, a co-treatment with chondrogenic factors such as bone morphogenic protein-7 is required.

The mechanism of the protective influence of Win on inflamed cartilage was presented in a recent study showing that dimethylbutyl-deoxy-delta-8-THC, a selective cannabinoid receptor 2 agonist, alleviates the expression of metalloproteinases [30]. Interestingly, in our in vitro study, we found that Win suppressed the expression of MMP-13 at lower concentrations, while a high concentration (1 µM) of Win promoted MMP-13 expression. Therefore, our results suggest the importance of the concentration when using cannabinoid agonists in treating osteoarthritis and also highlight the need for careful consideration when using the anti-inflammatory potential of medical marijuana to treat osteoarthritis. 

This study has several limitations. First, the cartilage samples tested here were derived from in vitro-expanded chondrocytes, which may not represent native cartilage. Second, the induction of inflammation in chondrocyte-derived cartilage used IL-1β at 10 ng/mL, which is a supraphysiological concentration. This treatment might result in severe damage to chondrocytes, masking the beneficial effects of Win treatment. Finally, we did not conduct a mechanistic study to explain the anti-inflammatory function of Win in cultured human chondrocytes. These limitations should be addressed in future studies. 

## 5. Conclusions

In this in vitro study, we demonstrated the mild protective effect of Win on the inflamed cartilage. However, whether cannabinoids can be used to treat joint diseases, such as osteoarthritis, requires further study in animals. 

## Data Availability

The datasets generated and/or analyzed during the current study are available from the corresponding author upon reasonable request.

## Supplementary Materials

The following supporting information can be downloaded at: https://www.mdpi.com/article/10.3390/biom13101502/s1. Table S1: Summary of donor information; Table S2: Primer sequences used for qRT-PCR.
