# Supplementary material for: Influence of the Synthetic Cannabinoid Agonist on Normal and Inflamed Cartilage: An In Vitro Study
Source: Biomolecules. 2023 Oct 10;13(10):1502. doi: 10.3390/biom13101502 (PMC10604475; doi:10.3390/biom13101502)
Supplement: Supplementary file 1 [file biomolecules-13-01502-s001.zip › biomolecules-2603577-supplementary.pdf]

# *Supplementary Material*

**Table S1. Summary of donor information**

| **No.** | **Patient ID** | **Age** | **Sex** | |
|---|---|---|---|---|
| **1** | 082218 | 66 | Female | Female Average<br><br>Age: 69.25 |
| **2** | 080718 | 67 | Female | |
| **3** | 020619 | 75 | Female | |
| **4** | 121019 | 69 | Female | |
| **5** | 110117 | 66 | Male | Male Ave<br><br>Age: 68.75 |
| **6** | 073118 | 67 | Male | |
| **7** | 082118 | 72 | Male | |
| **8** | 011920 | 70 | Male | |

**Table S2. Primer sequences used for qRT-PCR.**

| | |
|---|---|
| *SOX9(F)* | *GGCGGAGGAAGTCGGTGAAGAA* |
| *SOX9(R)* | *GCTCATGCCGGAGGAGGAGTGT* |
| *COL2(F)* | *GGATGGCTGCACGAAACATACCGG* |
| *COL2(R)* | *CAAGAAGCAGACCGGCCCTATG* |
| *AGG(F)* | *AGTCACACCTGAGCAGCATC* |
| *AGG(R)* | *AGTTCTCAAATTGCATGGGGTGTC* |
| *COL10(F)* | *CCCTCTTGTTAGTGCCAACC* |
| *COL10(R)* | *AGATTCCAGTCCTTGGGTCA* |
| *NF-kB(F)* | *AACAGAGAGGATTTCGTTTCCG* |
| *NF-kB(R)* | *TTTGACCTGAGGGTAAGACTTCT* |
| *IL-6(F)* | *ACTCACCTCTTCAGAACGAATTG* |
| *IL-6(R)* | *CCATCTTTGGAAGGTTCAGGTTG* |
| *MMP13(F)* | *ATGCAGTCTTTCTTCGGCTTAG* |
| *MMP13(R)* | *ATGCCATCGTGAAGTCTGGT* |
| *RPL13a(F)* | *CATAGGAAGCTGGGAGCAAG* |
| *RPL13a(R)* | *GCCCTCCAATCAGTCTTCTG* |
